# Intravitreal administration of multipotent mesenchymal stromal cells triggers a cytoprotective microenvironment in the retina of diabetic mice

**DOI:** 10.1186/s13287-016-0299-y

**Published:** 2016-03-16

**Authors:** Marcelo Ezquer, Cristhian A. Urzua, Scarleth Montecino, Karla Leal, Paulette Conget, Fernando Ezquer

**Affiliations:** Centro de Medicina Regenerativa, Facultad de Medicina Clínica Alemana-Universidad del Desarrollo, Av. Las Condes 12438, Lo Barnechea, Santiago, 7710162 Chile; Departamento de Oftalmología, Facultad de Medicina, Universidad de Chile, Av. Independencia 1027, Santiago, Chile

**Keywords:** Diabetes, Diabetic retinopathy, Multipotent mesenchymal stromal cells, Mesenchymal stem cells, Microenvironment, Cytoprotection, Retinal ganglion cells, Prevention

## Abstract

**Background:**

Diabetic retinopathy is a common complication of diabetes and the leading cause of irreversible vision loss in the Western world. The reduction in color/contrast sensitivity due to the loss of neural cells in the ganglion cell layer of the retina is an early event in the onset of diabetic retinopathy. Multipotent mesenchymal stromal cells (MSCs) are an attractive tool for the treatment of neurodegenerative diseases, since they could differentiate into neuronal cells, produce high levels of neurotrophic factors and reduce oxidative stress. Our aim was to determine whether the intravitreal administration of adipose-derived MSCs was able to prevent the loss of retinal ganglion cells in diabetic mice.

**Methods:**

Diabetes was induced in C57BL6 mice by the administration of streptozotocin. When retinal pro-damage mechanisms were present, animals received a single intravitreal dose of 2 × 10^5^ adipose-derived MSCs or the vehicle. Four and 12 weeks later we evaluated: (a) retinal ganglion cell number (immunofluorescence); (b) neurotrophic factor levels (real-time quantitative polymerase chain reaction (RT-qPCR) and enzyme-linked immunosorbent assay (ELISA)); (c) retinal apoptotic rate (TUNEL); (d) retinal levels of reactive oxygen species and oxidative damage (ELISA); (e) electrical response of the retina (electroretinography); (f) pro-angiogenic and anti-angiogenic factor levels (RT-qPCR and ELISA); and (g) retinal blood vessels (angiography). Furthermore, 1, 4, 8 and 12 weeks post-MSC administration, the presence of donor cells in the retina and their differentiation into neural and perivascular-like cells were assessed (immunofluorescence and flow cytometry).

**Results:**

MSC administration completely prevented retinal ganglion cell loss. Donor cells remained in the vitreous cavity and did not differentiate into neural or perivascular-like cells. Nevertheless, they increased the intraocular levels of several potent neurotrophic factors (nerve growth factor, basic fibroblast growth factor and glial cell line-derived neurotrophic factor) and reduced the oxidative damage in the retina. Additionally, MSC administration has a neutral effect on the electrical response of the retina and did not result in a pathological neovascularization.

**Conclusions:**

Intravitreal administration of adipose-derived MSCs triggers an effective cytoprotective microenvironment in the retina of diabetic mice. Thus, MSCs represent an interesting tool in order to prevent diabetic retinopathy.

**Electronic supplementary material:**

The online version of this article (doi:10.1186/s13287-016-0299-y) contains supplementary material, which is available to authorized users.

## Background

Diabetic retinopathy (DR) is one of the most common and frightening complications in patients with diabetes mellitus (DM) [1] and the leading cause of irreversible vision loss in developed countries [2]. In the Western world, the prevalence of DR in patients with DM is around 28 %, and strongly increases with the progression of DM [3]. Hence, almost all patients with Type 1 DM and 60 % of those with Type 2 DM will have some degree of DR after 20 years of DM evolution [4, 5].

Through the years, DR has been recognized primarily as a vascular disorder that involves pericyte loss, basement membrane thickening and endothelial dysfunction involving loss of retinal barrier integrity which leads to hemorrhage, vascular obliteration and the resulting neovascularization [6]. These events subsequently cause fibrovascular proliferation and blindness [6]. Nevertheless, it has been well documented that the hyperglycemic state adversely affects the entire neurosensory retina, and accelerates neuronal apoptosis [7, 8]. Thus, today DR is also considered a sensory neuropathy [9].

Although the molecular mechanisms by which DM induces neuronal degeneration and dysfunction are still not well understood, the role of hyperglycemia and the generation of reactive oxygen species (ROS) due to its exacerbating metabolism seem to be the most influential factor [10, 11]. ROS are mainly produced in the mitochondria through the electron transport chain, and it is well known that elevated ROS levels affect the survival and function of retinal neurons [12]. Excessive production of ROS results in the oxidative damage of several biomolecules including lipids, proteins and DNA. Retinal ganglion cells (RGCs) and glial cells are particularly sensitive to ROS-induced damage, which leads to the early death of these cells by apoptosis [13]. Additionally, it has been reported that increased ROS levels reduce the retinal levels of neurotrophic factors, including nerve growth factor (NGF), brain-derived neurotrophic factor (BDNF) and glial cell line-derived neurotrophic factor (GDNF), accelerating neuronal death [10, 12].

Nowadays, available treatments for DR are applicable in advanced stages of the disease and are primarily intended to regulate vascular changes principally mediated by the action of vascular endothelial growth factor (VEGF). These include laser treatment to destroy the hypoxic retinal cells that produce VEGF or the intravitreal administration of anti-VEGF drugs [14]. However, these therapies have only targeted the vascular pathology and have achieved limited success [15]. Therefore, the future generation of therapies that, applied at early stages of DR, can target the neural tissue eliciting a better visual prognosis are highly desirable.

Recently, the use of stem cells for the management of eye diseases has generated considerable interest [16]. Among the different type of stem cells, multipotent mesenchymal stromal cells (MSCs), also referred to as mesenchymal stem cells, appear as an ideal candidate for a cell therapy for DR because: (a) they can be obtained from different sources without major complications [17]; (b) they can be easily ex-vivo expanded [17]; (c) they can differentiate into neural cells or perivascular cells [18, 19], replacing the cells that are damaged during the course of DR; (d) they are able to secrete high levels of potent neuroprotective factors, including NGF, BDNF and GDNF [20, 21] that can reduce the apoptosis of neuronal cells in the retina; (e) they efficiently scavenge ROS, reducing the oxidative damage of the target tissues [22, 23]; and (f) MSCs have been used in cell therapy strategies to treat patients with different diseases, providing favorable outcomes without significant side effects [24, 25].

Previous work has demonstrated that the intravitreal injection of MSCs can help to ameliorate and repair different retinopathic injuries, mainly by the stabilization of retinal microvasculature [26, 27].

In this study, we evaluated whether an intravitreal administration of adipose-derived MSCs was able to prevent the loss of RGCs in diabetic mice. For this, severe diabetes was induced in C57/BL6 mice by the administration of a single high dose (200 mg/kg) of streptozotocin (STZ) [28, 29]. Diabetes progression was evaluated according to the levels of glucose, insulin and glycated hemoglobin in blood samples. Twelve weeks after diabetes induction, animals were randomly assigned into two groups: one group received a single intravitreal administration of 2 × 10^5^ adipose-derived MSCs (DM + MSC mice) and the other group received the vehicle (DM mice). In addition, a third group of nondiabetic animals (Normal mice) was included as control. Four and 12 weeks post-MSC administration, we evaluated: (a) RGC number by immunofluorescence; (b) the levels of neuroprotective factors by real-time quantitative polymerase chain reaction (RT-qPCR) and enzyme-linked immunosorbent assay (ELISA); (c) retinal apoptotic rate by TUNEL; (d) retinal ROS levels and oxidative damage by ELlSA; (e) electrical response of the retina by electroretinography; (f) pro-angiogenic and anti-angiogenic factors levels by RT-qPCR and ELISA; and (g) retinal blood vessels by angiography. Furthermore, 1, 4, 8 and 12 weeks post-MSC administration, the presence of donor cells in the retina and their differentiation into neural and perivascular-like cells were assessed by immunofluorescence and flow cytometry.

We found that the intravitreal administration of MSCs increases neurotrophic factor levels, reduces oxidative damage of the retina and prevents RGC loss in diabetic mice.

## Methods

### Animals

C57BL/6 and C57BL76-Tg(ACTB-EGFP)1Obs mice (Jackson Laboratory, Bar Harbor, ME, USA) were housed at constant temperature and humidity, with a 12:12 hour light–dark cycle and unrestricted access to standard diet and water. When required, animals were lightly anesthetized with sevofluorane (Abbot, Tokyo, Japan) or deeply anesthetized with ketamine (Drag Pharma, Santiago, Chile) plus xylazine (Centrovet, Santiago, Chile). All animal protocols used were approved by the Ethic Committee of Facultad de Medicina Clínica Alemana-Universidad del Desarrollo.

### Diabetes induction

Ten-week-old male C57BL/6 mice were lightly anesthetized and received an intraperitoneal injection of 200 mg/kg STZ (Calbiochem, La Jolla, CA, USA) immediately after dissolving it in 0.1 M citrate buffer pH 4.5 (DM mice), or citrate buffer only (Normal mice). It has been reported that this protocol of STZ administration causes a massive cytotoxic destruction of beta-pancreatic cells generating a condition of severe hyperglycemia, which accelerates the appearance of the secondary complications associated with diabetes [28, 30].

### Blood glucose quantification

Blood samples were collected from the tail vein of non-fasted alert animals, and glucose levels were determined with the glucometer system Accu-Chek Performa from Roche Diagnostic (Mannheim, Germany).

### Plasma insulin quantification

Blood samples were collected from the tail vein of fasted alert animals. Plasma was recovered by centrifugation, and insulin concentrations were measured using a mouse insulin ultrasensitive ELISA kit (Mercodia, Uppsala, Sweden).

### Glycated hemoglobin quantification

Blood samples were collected from the tail vein of fasted alert animals, and HbA_1c_ percentages were assessed using the DCA2000 analyzer (Bayer Corporation, Pittsburgh, PA, USA) as previously described [31].

### Isolation, ex vivo expansion and characterization of MSCs

Eight- to 10-week-old female C57BL/6 or C57BL/6-Tg(ACTB-EGFP)1Obs mice were sacrificed by cervical dislocation. Epididymal fat was dissected, washed with phosphate-buffered saline (PBS) and cut into small pieces. Tissues were then digested with 1 mg/mL collagenase type II (Gibco, Grand Island, NY, USA) in PBS and incubated with agitation at 37 °C for 2 hours. At the end of digestion, 10 % fetal bovine serum (FBS; Gibco, Auckland, New Zealand) was added to neutralize collagenase. The mixture was then centrifuged at 400 g for 10 minutes to remove floating adipocytes. Pellets were re-suspended in α-minimum essential medium (α-MEM; Gibco) supplemented with 10 % FBS and 0.16 mg/mL gentamicin (Sanderson Laboratory, Santiago, Chile), plated at a density of 7000 cells/cm^2^ and cultured at 37 °C in a 5 % CO_2_ atmosphere. When foci reach confluence, cells were detached with 0.25 % trypsin, 2.65 mM EDTA (Sigma-Aldrich, St. Louis, MO, USA), centrifuged and subcultured at 7000 cells/cm^2^. After two subcultures, cells were characterized according to their adipogenic and osteogenic differentiation potential. For this, MSCs were incubated with standard adipogenic or osteogenic differentiation media for 14 and 21 days, respectively. To evaluate the adipogenic potential, cultures were stained with Oil Red (Sigma-Aldrich). To evaluate the osteogenic potential, cultures were fixed with 10 % formaldehyde and stained with Alizarin Red (Sigma-Aldrich) as previously described [28]. Immunophenotyping was performed by flow cytometry analysis after immunostaining with monoclonal antibodies against the putative murine MSC markers α-SMA (FITC-conjugated; BD Bioscience, San Jose, CA, USA), Sca-1 (PE-conjugated; eBioscience, San Diego, CA, USA), and CD90 (PECy7-conjugated; eBioscience), or characteristic markers of hematopoietic cell lineages CD45 (AF780-conjugated; BD Bioscience) and CD11b (PECy5-conjugated; eBioscience).

### Intravitreal administration of MSCs

Twelve weeks after DM induction, mice were lightly anesthetized and 0.5 % proparacaine (Alcon, Santiago Chile) was topically applied. A cell suspension containing 2 × 10^5^ MSCs, passage 2, in 2 μL saline (DM + MSC mice), or 2 μL saline (DM mice) was slowly injected into the vitreous cavity through the pars plana using a 33-gauge microsyringe (Hamilton, Reno, NV, USA). Eyes showing massive vitreous hemorrhaging after the injection were excluded from the study.

### Quantification of RGCs

Animals were euthanatized by cervical dislocation and eyes were enucleated and fixed in 4 % paraformaldehyde (Merck, Darmstadt, Germany). Fixed eyes were orientated to permit radial sectioning and embedded in paraffin. Eyes were sectioned (4 μm), and sections were deparaffinized and incubated with rat anti-mouse beta-3-tubulin antibody (marker of RGCs) (Santa Cruz Biotechnology, Dallas, TX, USA). Afterwards, sections were washed and incubated with anti-rat-Alexa555 secondary antibody (Vector Labs, Burlingame, CA, USA) and counterstained with 4’-6’-diamidino-2-phenylindole (DAPI; Invitrogen, Grand Island, NY, USA). Only sections that included a full length of retina approximately along the horizontal meridian, passing through the ora serrata and the optic nerve in both the temporal and nasal hemispheres were used. The number of RGCs in the ganglion cell layer was quantified by counting labeled cells from the temporal to the nasal ora serrata in five serial sections using the Fluoview FV10i confocal microscope (Olympus, Tokyo, Japan). Samples were blind-analyzed by two independent observers. Data were presented as number of RGCs per 100 μm of retina length [32].

### Detection of donor MSCs^GFP^

For in situ detection of MSCs^GFP^, eyes were fixed in 4 % paraformaldehyde. One day later, they were embedded in paraffin and radially sectioned. Four-micrometer-thick sections were deparaffinized, incubated with rabbit anti-GFP antibody (eBioscience) at 4 °C overnight, incubated with goat anti-rabbit-FITC antibody (Vector Labs) at room temperature for 1 hour, and counterstained with DAPI. Sections were examined with the Fluoview FV10i confocal microscope.

For the quantification of MSCs^GFP^, eyes were washed twice with ice-cold PBS, chopped and digested with 1 mg/mL collagenase type II at 37 °C for 30 minutes. Collagenase was inactivated with 10 % FBS and cell suspensions were filtered through a 100-μm strainer and washed twice with ice-cold PBS. To ensure MSC^GFP^ recognition, cells in the suspension were fixed and permeabilized with BD Cytofix/Cytoperm kit (BD Pharmingen, San Jose, CA, USA) and suspended in 1 mL PBS with 2 % FBS plus 1 μL undiluted anti-GFP AlexaFluor647 antibody (Molecular Probes, Grand Island, NY, USA). After incubation at 4 °C for 12 hours, cells were washed, filtered through a 30-μm mesh and acquired in a CyAn ADP flow cytometer (DakoCytomation, Carpinteria, CA, USA) as previously described [33]. Data were analyzed with Summit v4.3 software. Criteria used to consider an event as an MSCs^GFP^ were forward scatter and side scatter similar to ex vivo expanded MSCs and positive fluorescence both in FL1 (GFP) and FL8 (anti-GFP AlexaFluor647) channels. Eyes from untreated diabetic mice were used as autofluorescence controls. Results were presented as number of MSCs^GFP^ per eye.

### Evaluation of MSCs^GFP^ differentiation into neural-like or perivascular-like cells

To determine whether donor MSCs differentiated into neural-like cells, eye sections were co-stained with rabbit anti-GFP antibody (eBioscience) and rat anti-mouse beta-III-tubulin antibody (marker of RCGs) (Santa Cruz Biotechnology) or were co-stained with rabbit anti-GFP antibody and rat anti-mouse GFAP antibody (marker of retinal astrocytes) (Santa Cruz Biotechnology). Afterwards, sections were washed and incubated with anti-rabbit-FITC and anti-rat-Alexa555 secondary antibodies (Vector Labs) and counterstained with DAPI.

To determine whether donor MSCs differentiated into perivascular-like cells, eyes were fixed for 2 hours in 4 % paraformaldehyde. Afterwards, the cornea and lens were removed and the retina was dissected and flattened onto silanized glass slides facilitated by four equidistant radial cuts into the peripheral retina. Wholemounts were permeabilized with 1 % digitonin (Calbiochem) in PBS at room temperature for 1 hour and immunohistochemically stained by overnight incubation with rabbit anti-GFP antibody and rat anti-mouse NG2 antibody (marker of pericytes) (Millipore, Darmstadt, Germany) at 4 °C. Samples were also incubated with Isolectin GS-IB4 from *Griffonia simplicifolia* conjugated to AlexaFluor647 (Life Technology, Grand Island, NY, USA) to allow the detection of retinal capillaries. Afterwards, samples were washed and incubated with anti-rabbit-FITC and anti-rat-Alexa555 secondary antibodies (Vector Labs).

In all cases, retinal tissues without exposure to the primary antibodies were used as controls for immunostaining. Samples were analyzed under confocal microscopy by taking optical sections of 1 μm. Data were analyzed with the Olympus FV10-ASW2.1 software.

### Quantification of mRNA levels of neurotrophic, pro-angiogenic and anti-angiogenic factors

Eyes were enucleated and washed two times with ice-cold PBS. Total RNA was purified using Absolutely RNA Miniprep kit (Stratagene, Santa Clara, CA, USA). One microgram of total RNA was used for reverse transcription. RT-PCR reactions were performed in a final volume of 10 μL containing 50 ng cDNA, PCR LightCycler-DNA Master SYBERGreen reaction mix (Roche, Indianapolis, IN, USA), 3 mM MgCl_2_ and 0.5 μM of the primers for the amplification of NGF, basic fibroblast growth factor (bFGF), GDNF, BDNF, ciliary neurotrophic factor (CNTF), VEGF-α, platelet-derived growth factor (PDGF), angiopoietin 1 (ANG-1) and thrombospondin-1 (TSP-1) (Additional file 1: Table S1), using a Light-Cycler 1.5 thermocycler (Roche). To ensure that amplicons were from mRNA and not for genomic DNA amplifications, controls without reverse transcription were included. Amplicons were characterized according to their size evaluated by agarose gel electrophoresis and to their melting temperature determined in the LightCycler thermocycler. Relative quantifications were performed by the ΔΔCT method. The mRNA level of each target gene was standardized against the mRNA level of GAPDH, for the same sample. Results were presented as fold-change versus normal mice.

### Quantification of protein levels of neurotrophic and anti-angiogenic factors

Eyes were enucleated and washed two times with ice-cold PBS. Samples were mechanically lysed in lysis buffer (RayBiotech, Norcross, GA, USA) containing a protease inhibitor cocktail (Thermo, Waltham, MA, USA) and centrifuged at 12,000 g for 10 minutes. The levels of NGF, bFGF, GDNF and TSP-1 were measured in the supernatant of the lysates using the Mouse beta-NGF ELISA kit (RayBiotech), Mouse FGF basic Quantikine ELISA kit (R&D Systems, Minneapolis MN, USA), Mouse GDNF ELISA kit (MyBioSource, San Diego, CA, USA) and Mouse thrombospondin 1 ELISA kit (MyBioSource), respectively. Data were normalized per mg of protein present in each sample.

### Quantification of apoptotic cells

The presence of apoptotic cells in the retina was evaluated by the TUNEL technique in 4-μm thick sections of the eye, using the In Situ Cell Death Detection Kit (Roche) following the manufacturer’s instructions. In each experiment, adjacent sections incubated without TdT served as negative control. Nuclei were counterstained with DAPI and samples were observed by confocal microscopy. Each section was scanned systematically from the temporal to the nasal ora serrata looking for fluorescent cells indicative of apoptosis. The number of TUNEL-positive cells per section was determined using the Olympus FV10-ASW2.1 software, and the apoptosis rate was expressed as fold-change versus normal mice.

### Quantification of ROS

Retinas were carefully dissected and mechanically lysed in lysis buffer (RayBiotech) containing a protease inhibitor cocktail. For quantification of ROS level, equal volumes of retinal lysates were incubated with 10 μmol/L 2,7-dichloro-dihydro-fluorescein diacetate (H_2_DCFDA; Invitrogen) for 1 hour at 37 °C. Fluorescence was measured in a fluorimeter (Turner, Sunnyvale, CA, USA) with excitation of 485 nm and emission of 520 nm as previously reported [34]. Data were normalized per mg of protein present in each sample and expressed as fold-change versus normal mice.

### Quantification of oxidative damage

Lipid peroxidation was determined by a method that measures the amount of thiobarbituric acid reactivity by the amount of malondialdehyde (MDA) formed during acid hydrolysis of the lipid peroxide compounds using the Lipid Peroxidation MDA Assay kit (Cayman, Ann Arbor, MI, USA). Retinas were mechanically lysed in lysis buffer (RayBiotech) containing a protease inhibitor cocktail and the antioxidant butyl hydroxytoluene (BHT). Lipid peroxidation was quantified sprectophotometrically at 540 nm (Thermo spectronic). MDA level was normalized per mg of protein present in each sample.

Protein nytrosilation was quantified in retinal lysates containing a protease inhibitor cocktail, measuring nitrotyroxine level with the 3-Nitrotyrosine ELISA kit (Abcam, Cambridge, UK) following the manufacturer’s instructions. The nitrotyroxine level was normalized per mg of protein present in each sample.

DNA oxidation was measured in genomic DNA isolated from retinal samples by DNAzol (Invitrogen). DNA was digested by incubation with DNAsa RQ1 (Promega, Madison, WI, USA) and 8-OHdG levels were quantified using the Oxidative DNA damage ELISA kit, following the manufacturer’s instructions. 8-OHdG levels were normalized per μg of DNA present in each sample.

For all oxidative markers, data were expressed as fold-change versus normal mice.

### Evaluation of retinal electrical response

Evaluation of retinal function was carried out by electroretinography (ERG) using the Handheld Multi-species ElectroRetinoGraph Instrument (Ocuscience, Kansas City, MO, USA). In brief, the animals were prepared under red light illumination, anesthesia was provided by the inhaled administration of sevoflurane and pupils were dilated by using tropicamide acetate (Alcon). Body temperature was maintained at 37 °C with a heating plate. All animals recovered from anesthesia after the procedure.

Previous to the measurements, mice were maintained for a period of dark adaptation of 16 hours. The electrical responses were registered by using a corneal electrode with methylcellulose as a coupling agent. Reference and ground electrodes were placed in the ear and in the tail, respectively. Stimulation and parameter recording were carrying out as defined by the international Society for Clinical Electrophysiology of Vision (ISCEV). In brief, flash stimuli were as follows: 0.01 and 3.0 cd · s · m^–2^ in scotopic and photopic conditions. The a-wave amplitude was measured from the baseline to the trough of the a-wave and the b-wave amplitude was measured from the trough of the a-wave to the peak of the b-wave as previously reported [35]. Oscillatory potentials (OPs) were isolated by treating measurements with a digital bandpass filter between 60 and 200 Hz, after dark adaptation using 3.0 cd · s · m^–2^ flash stimuli. The peak amplitudes of the first six waves were recorded from baseline, as previously described [36].

### Quantification of retinal vasculature and detection of vascular leakage

Animals were lightly anesthetized and injected in the tail vein with 0.2 mL saline containing 10 mg of 2 × 10^6^ molecular weight fluorescein-dextran (Sigma-Aldrich). Five minutes later, animals were euthanatized by cervical dislocation and eyes were enucleated and fixed in 4 % paraformaldehyde for 2 hours. Afterwards, the cornea and lens were removed, and the retina was dissected and flattened onto glass slides facilitated by four equidistant radial cuts into the peripheral retina. The flat-mounted retinas were photographed by confocal microscopy and at least 15 images per animal were analyzed. The fluorescent area, representative of the retinal vascular area, of each image was quantified using the Image J 1.34 software (NIH, Bethesda, USA). Data were presented as retinal vascular area/total retinal area. Retinal vascular leakage was determined by the presence of areas of extravasated FITC-dextran.

### Statistical analysis

Data were presented as mean ± standard error of the mean (SEM). Multiple group comparisons were performed by analysis of variance (ANOVA) followed by Bonferroni post-hoc test, while comparisons between two experimental group were performed by Student’s *t* test. *p* < 0.05 were considered statistically significant.

## Results

### Diabetes induction and retinopathy condition

**Fig. 1:**
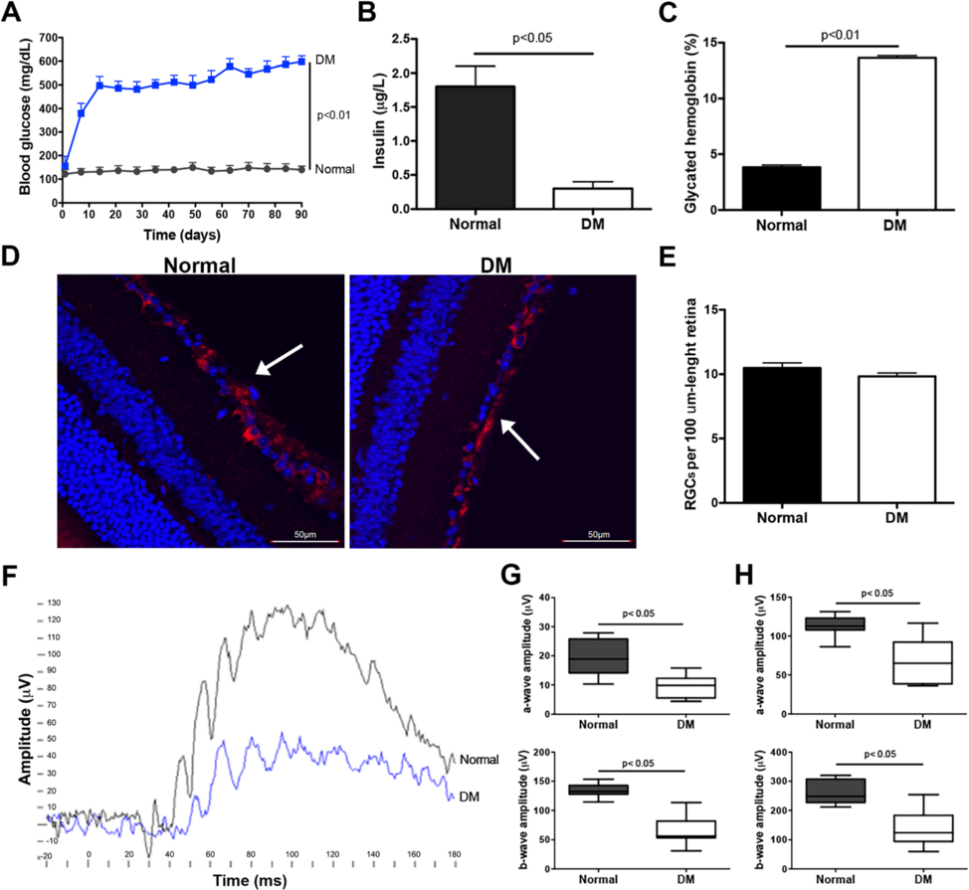
Characterization of diabetes mellitus (*DM*) stage and DR development prior to MSC administration. C57BL/6 adult male mice were injected either with 0.1 M citrate buffer (Normal mice) or 200 mg/kg STZ in 0.1 M citrate buffer (DM mice). **a** Blood glucose level was determined weekly in venous blood samples obtained from alert non-fasted animals. **b** Insulinemia and **c** glycated hemoglobin levels were determined 12 weeks after SZT administration. Retinal histology was analyzed 12 weeks after SZT administration in serial 4-μm eye sections, immunolabeled with β3-tubulin antibody (marker of retinal ganglion cells (*RGCs*)). **d** Samples were observed under confocal microscopy focusing on the ganglion cell layer of the retina (*arrows*). **e** RGCs in the ganglion cell layer of the retina were quantified. **f** Representative ERG showing a characteristic decreases in a-wave and b-wave in DM mice in comparison to normal mice using a stimulus of 0.01 cd · s · m^–2^. *Black line*, normal mice; *blue line*, DM mice. The amplitudes of a-waves and b-waves recorded under scotopic conditions using stimuli of **g** 0.01 cd · s · m^–2^ or **h** 3.0 cd · s · m^–2^ were quantified. Qualitative data are representative of eight eyes per group. Quantitative data correspond to mean ± SEM of eight eyes per group

To induce DM, C57BL6 mice were treated with a single dose of 200 mg/kg STZ, since this protocol causes a rapid and massive destruction of pancreatic beta cells [28]. Diabetic mice were maintained without insulin supplementation to allow for the progression of severe diabetes and the appearance of its complications, including DR. Ten days after STZ administration, blood glucose levels reached their highest concentration, which was three times higher than in normal individuals (Fig. 1a). Twelve weeks after DM induction, elevated blood glucose level was also correlated with a severe reduction in plasma insulin level and a marked increase in glycated hemoglobin level (Fig. 1b and c). At this time, DM mice maintained the same number of RGCs compared to age matched normal mice (Fig. 1d and e); however, retinal electrical response was altered. This could be evidenced by a significant reduction in the amplitudes of the a-wave and b-wave of the ERG of DM mice in comparison to normal mice under stimuli of 0.01 cd · s · m^–2^ and 3.0 cd · s · m^–2^ (Fig. 1f, g and h).

**Fig. 2:**
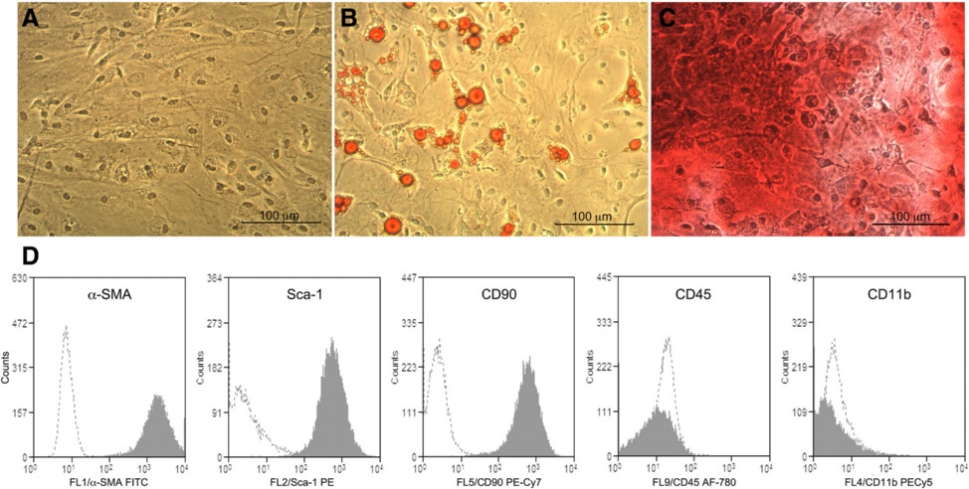
Characterization of adipose-derived MSCs isolated from adult C57BL/6 mice. **a** Isolated cells were cultured in α-MEM containing 10 % selected FBS into plastic dishes and differentiated into **b** adipogenic and **c** osteogenic lineages. Cells were also immunophenotyped according to the expression of the putative murine MSC markers alpha-SMA, SCA-1 and CD90 and the non-expression of the markers characteristic of hematopoietic cell lineages—CD45 and CD11b. **d** Open histograms represent cells stained with isotype controls; filled histograms represent cells labeled with specific antibodies. Data shown are representative of cells isolated from four different normal animals

Adipose-derived MSCs were isolated, expanded and characterized according to their adipogenic and osteogenic potential (Fig. 2a–c), and by the presence of the putative murine MSC markers α-SMA, SCA-1 and CD90 and by the non-expression of markers characteristics of hematopoietic cell lineages—CD45 and CD11b (Fig. 2d). Twelve weeks after diabetes induction, DM mice were randomly assigned into two groups: one group that received an intravitreal administration of 2 × 10^5^ MSCs (DM + MSC mice) and another group that received an intravitreal administration of the vehicle (DM mice). Additionally, we also maintained a group of normal non-diabetic mice (Normal mice) as a control.

#### MSC administration prevents RGCs loss

**Fig. 3:**
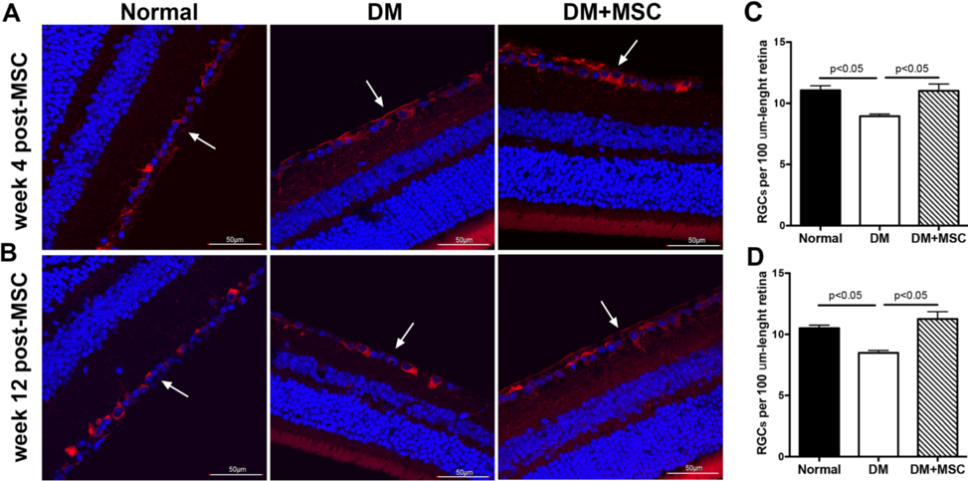
Mesenchymal stromal cell (*MSC*) administration prevents retinal ganglion cell (*RGC*) loss. Twelve weeks after STZ administration, diabetic mice received an intravitreal injection of 2 μL saline (*DM*) or 2 × 10^5^ MSCs resuspended in 2 μL saline (*DM + MSC*). Retinal histology was analyzed in serial 4-μm eye sections, immunolabeled with β3-tubulin antibody (marker of RGCs). Samples were observed under confocal microscopy focusing on the ganglion cell layer of the retina (*arrows*), **a** 4 weeks and **b** 12 weeks after MSC administration. RGCs in the ganglion cell layer of the retina were quantified at both experimental times (**c** and **d**). Qualitative data are representative of eight eyes per group and per analyzed time. Quantitative data correspond to mean ± SEM of eight eyes per group and per analyzed time

Since one of the earliest events in the onset of DR is the death of RGCs [13], we tested whether MSC administration was able to prevent the loss of these cells in the retina. As expected, 16 weeks after DM induction (4 weeks after vehicle administration) DM mice presented a significant reduction (~20 %) in the number of RGCs in the retina. However, MSC administration completely prevented this reduction (Fig. 3a and c). The preventive effect was maintained until at least 24 weeks after diabetes induction (12 weeks after MSC administration) when DM + MSC mice presented an equivalent number of RGCs as compared to cells in the normal mice (Fig. 3b and d).

#### Donor MSCs remains in the eye but do not differentiate into neural-like or perivascular-like cells

**Fig. 4:**
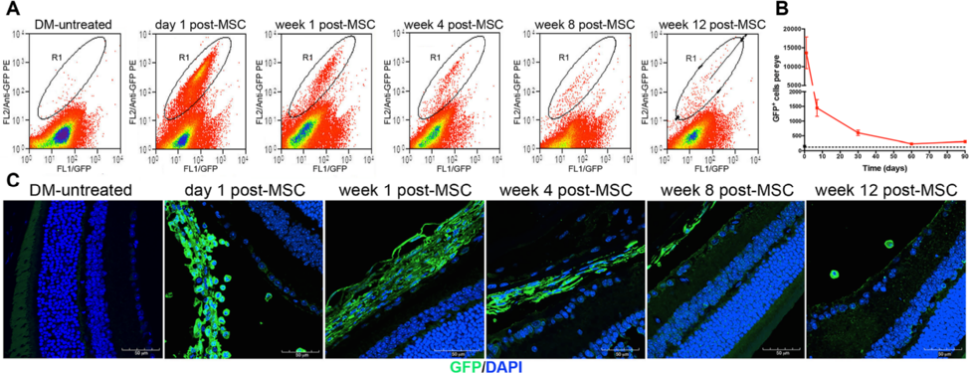
Donor mesenchymal stromal cells (*MSCs*) remain in the eye at least until 12 weeks after their administration. Twelve weeks after STZ administration, diabetic mice received an intravitreal dose of 2 × 10^5^ MSCs^GFP^ in 2 μL saline. On days 1, 4, 8 and 12 weeks later, the presence of donor cells was assessed in the eye by flow cytometry. **a** Events in gate R1 represent GFP^+^ cells. **b** Data were quantified, and the *dotted line* represents the detection limit. **c** Presence of donor cells in the eye was also corroborated by immunofluorescence. Qualitative data are representative of eight eyes per analyzed time. Quantitative data correspond to mean ± SEM of eight eyes per analyzed time. *DM* Diabetes mellitus, *GFP* green fluorescent protein

**Fig. 5:**
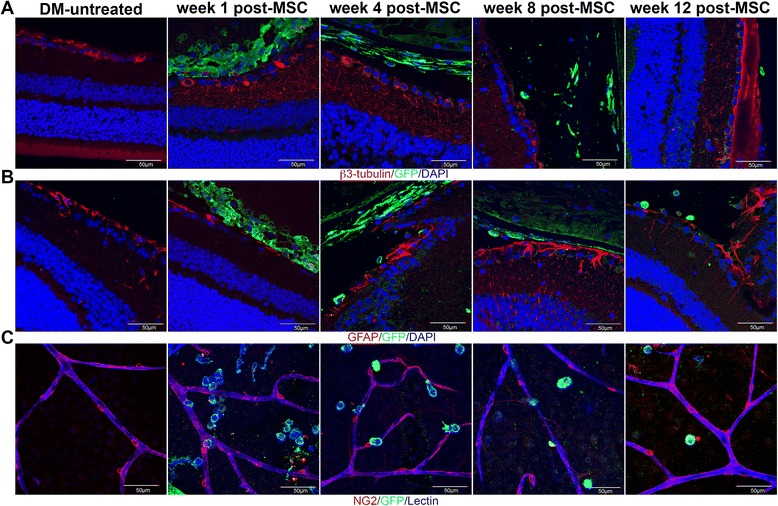
Most donor mesenchymal stromal cells (*MSCs*) do not differentiate into neural- or perivascular-like cells. On days 1, 4, 8 and 12 weeks after MSC administration, immunofluorescences were performed in eye sections to evaluate differentiation of MSCs into RGCs by **a** colocalization of green fluorescent protein (*GFP*) with the RGC marker β3-tubulin or **b** differentiation into retinal astrocytes by colocalization of GFP with the astrocyte marker GFAP. **c** To evaluate differentiation of MSCs into perivascular cells, immunofluorescences were performed to colocalize GFP with the pericyte marker NG2 in whole mount retinal preparations. Lectin was also added to detect blood vessels. Qualitative data are representative of six eyes per analyzed time

One possibility to explain the therapeutic effect observed is that the administered MSCs could differentiate into RGCs, replacing the damaged cells, or into another neural or perivascular cell type that could act as a support cell to prevent RGC death. To assess this possibility, first we evaluated whether administered MSCs could remain in the eye and integrate into the retina of DM mice. Using MSCs isolated from transgenic mice that constitutively express GFP, we observed by flow cytometry and immunohistofluorescence that the number of donor cells in the eyes were decreasing over time, but they were still detectable 12 weeks after their administration (Fig. 4). Most donor cells remained in the vitreous cavity and did not integrate into the retina (Fig. 4c). To evaluate whether MSCs could differentiate into neural or perivascular cells we tried to colocalize GFP with a marker of RGCs (β3-tubulin) (Fig. 5a), with a marker of retinal astrocytes (GFAP) (Fig. 5b) or with a marker of retinal pericytes (NG2) (Fig. 5c). In most cases, GFP -positive cells did not express any of these markers at any of the analyzed times. However, we also detected a small number of MSCs^GFP^ that also expressed the marker of RGC (Additional file 2: Figure S1A), the marker of astrocyte (Additional file 2: Figure S1B) or the marker of pericyte (Additional file 2: Figure S1C).

#### The intravitreal administration of MSCs increases the intraocular levels of neurotrophic factors

MSCs are known to produce, both in vitro and in vivo, a broad range of trophic factors that have been associated with tissue regeneration and neuronal survival [20]. To evaluate whether the therapeutic effects observed after MSC administration could be related to the generation of a pro-regenerative microenvironment, the intraocular levels of several neurotrophic factors were measured.

**Fig. 6:**
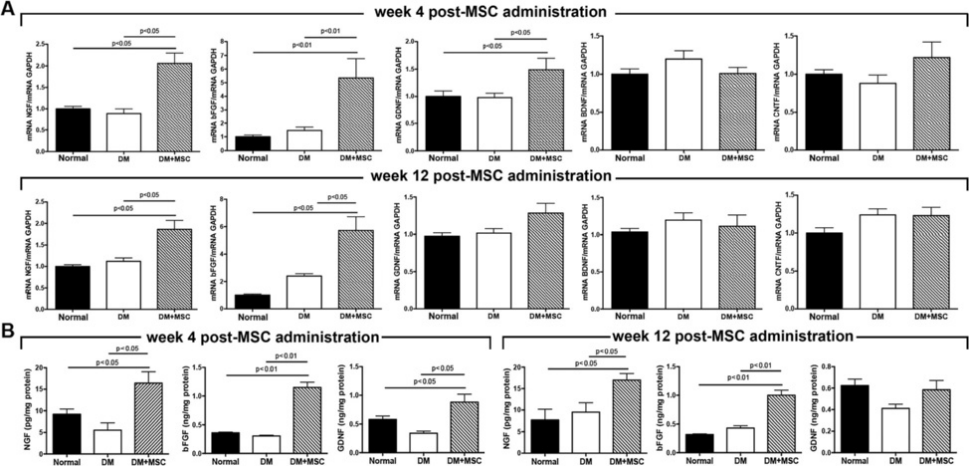
The intravitreal administration of mesenchymal stromal cells (*MSCs*) increases the intraocular levels of neurotrophic factors. Four and 12 weeks after the administration of MSCs or vehicle, the intraocular levels of several neurotrophic factors were measured by **a** RT-qPCR and **b** ELISA. Quantitative data correspond to mean ± SEM of eight eyes per group and per analyzed time. *BDNF* Brain-derived neurotrophic factor, *bFGF* basic fibroblast growth factor, *CNTF* ciliary neurotrophic factor, *DM* diabetes mellitus, *GDNF* glial cell line-derived neurotrophic factor, *NGF* nerve growth factor

Four weeks after MSC administration, we observed a significant increase in the mRNA levels of NGF, bFGF and GDNF in the eyes of DM + MSC mice compared to age-matched DM mice and normal mice (Fig. 6a). The mRNA levels of NGF and bFGF remained elevated in the eye of DM + MSC mice 12 weeks post-MSC administration (Fig. 6a). These neurotrophic factors were also elevated at protein levels at both experimental times (Fig. 6b).

One of the main mechanisms associated with the neuroprotective effect of neurotrophic factors is the inhibition of apoptosis. To evaluate whether the microenvironment generated by the secretion of trophic factors could reduce the apoptosis of RGCs, the apoptotic rate was determined by the TUNEL assay. Examination of retinas of diabetic mice showed a significant increase in TUNEL-positive cells both in DM and DM + MSC mice 4 and 12 weeks post-administration of vehicle or MSCs compared to age-matched normal mice (Additional file 3: Figure S2A–D). However, in all cases, apoptotic cells were present in the outer nuclear layer of the retina. At the experimental times evaluated, we could not find any TUNEL-positive cell in the ganglion cell layer of the retina.

#### The intravitreal administration of MSCs reduces the retinal oxidative damage

**Fig. 7:**
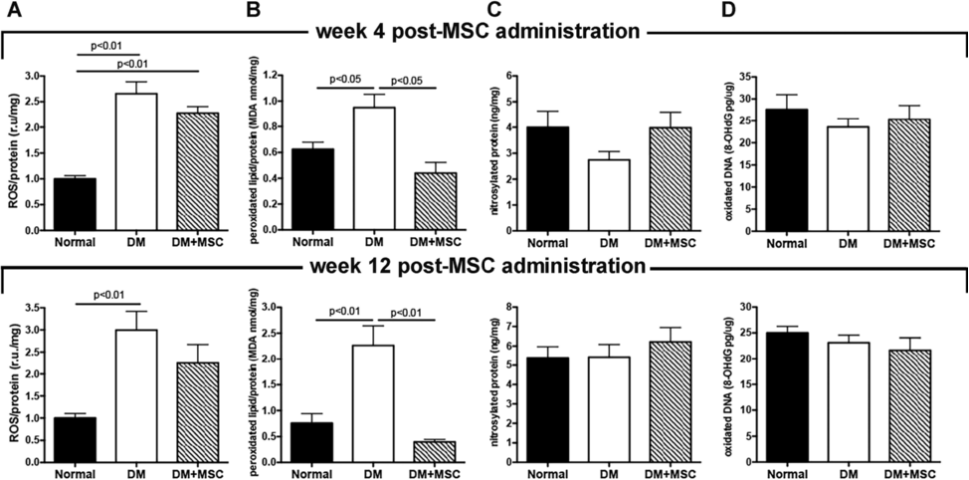
The intravitreal administration of mesenchymal stromal cells (*MSCs*) reduces the retinal oxidative damage. Four and 12 weeks after the administration of MSCs or vehicle the retinas were dissected and the levels of **a** reactive oxygen species (*ROS*), **b** lipid peroxidation, **c** protein nitrosylation and **d** DNA oxidation were measured. Quantitative data correspond to mean ± SEM of eight eyes per group and per analyzed time. *DM* Diabetes mellitus, *MDA* malondialdehyde

Oxidative and nitrative modifications of retinal macromolecules occur promptly in the course of DR and are associated with the early death of retinal neurons [37, 38]. It has been postulated that MSCs could efficiently scavenge reactive species [22]. To evaluate the effect of MSC administration on the oxidative damage of the retina, we measured total ROS level and different markers of oxidative damage 4 and 12 weeks after MSC administration. As expected, diabetes induced a significant increase in the total amount of oxidative species in the retina at both experimental times compared with the oxidative species in age-matched normal mice (Fig. 7a). The elevated ROS levels correlated with a significant increase in the lipid peroxidation level in the retina (Fig. 7b), but were not enough to induce detectable oxidative damage at the protein and DNA levels (Fig. 7c and d). MSC administration induced a small non-significant reduction in ROS levels in the retina (Fig. 7a), but a strong reduction in lipid peroxidation levels at both experiential times, reaching values similar to those observed in normal mice (Fig. 7b).

#### The intravitreal administration of MSCs has a neutral effect in the electrical response of the retina in DM mice

**Fig. 8:**
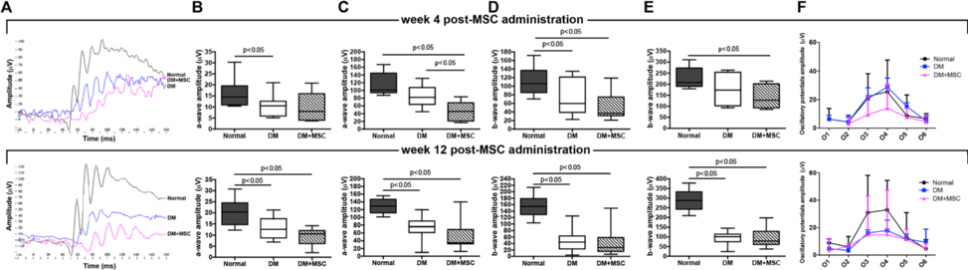
The intravitreal administration of mesenchymal stromal cells (*MSCs*) has neutral effect in the electrical response of the retina in diabetes mellitus (*DM*) mice. Four and 12 weeks after the administration of MSCs or vehicle, the electrical responses of the retina were measured by ERG. **a** Representative ERG showed decreases in a-wave and b-wave in DM mice and in DM + MSC mice in comparison to normal mice using a stimulus of 0.01 cd · s · m^–2^; *black line*, normal mice; *blue line*, DM mice; *purple line*, DM + MSC mice. The a-waves were quantified under scotopic conditions using stimuli of **b** 0.01 cd · s · m^–2^ or **c** 3.0 cd · s · m^–2^. The b-waves were quantified under scotopic conditions using stimuli of **d** 0.01 cd · s · m^–2^ or **e** 3.0 cd · s · m^–2^. **f** The peak amplitudes of the first six waves of the oscillatory potentials were quantified. Qualitative data are representative of eight eyes per group and per analyzed time. Quantitative data correspond to mean ± SEM of eigth eyes per group and per analyzed time

It is well known that prior to the development of any histologically detectable retinal alteration, diabetic retinas display a significant decrease in neuronal function that could be evidenced by ERG [39]. Thus, we evaluated whether MSC administration could improve the electrical response in the retina of DM mice. For this we performed dark-adapted ERG, 4 and 12 weeks after MSC administration and, using stimuli of 0.01 cd · s · m^–2^ and 3.0 cd · s · m^–2^, we observed that no matter whether diabetic mice were treated with MSCs or only the vehicle, they had a significant reduction in the a-wave and b-wave amplitudes compared to age-matched normal mice (Fig. 8a–e). We also measured Ops, since it has been previously reported that OPs are altered in diabetic individuals [40], and OPs are likely due to inner retinal neurotransmission, which represents the synaptic activity between amacrine neurons and RGCs [41]. There were no significant differences in the amplitudes of the OPs between the experimental groups at both experimental times analyzed (Fig. 8f).

#### The intravitreal administration of MSCs does not induce a provasculogenic microenvironment in the retina

While the growth of new blood vessels is beneficial in some ischemic conditions like ischemic heart disease [42], neovascularization in most ocular diseases, including DR, is devastating to visual function [6]. It is well known that MSCs are able to secrete, in addition to neurotrophic factors, strong angiogenic factors [43]. This could be a major limitation since MSC administration might worsen DR.

**Fig. 9:**
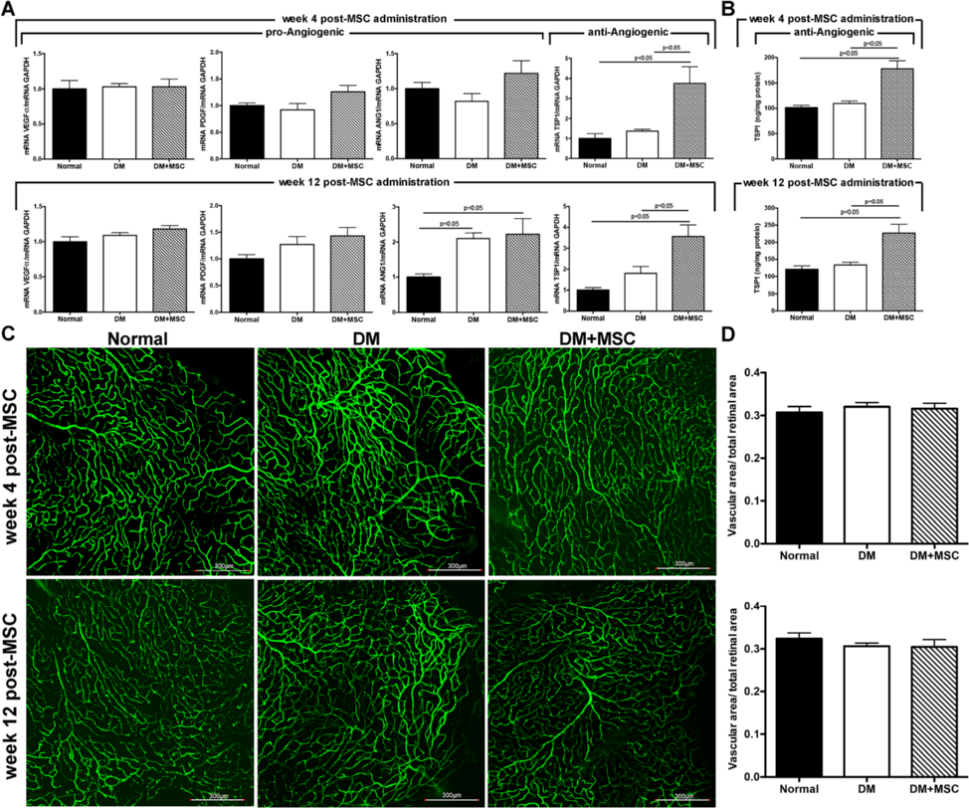
The intravitreal administration of mesenchymal stromal cells (*MSCs*) does not induce a provasculogenic microenvironment in the retina. Four and 12 weeks after the administration of MSCs or vehicle, the intraocular levels of pro-angiogenic and anti-angiogenic factors were measured by **a** RT-qPCR and **b** ELISA. Retinal blood vessels were visualized at both experimental times by the administration of FITC-dextran in the tail vein. **c** Retinas were dissected, flat mounted and observed by confocal microscopy. **d** Retinal vasculature was quantified and expressed as vascular area/total retinal area. Quantitative data correspond to mean ± SEM of eight eyes per group and per analyzed time. Qualitative data are representative of eight eyes per group and per analyzed time. *ANG1* Angiopoietin 1, *DM* diabetes mellitus, *PDGF* platelet-derived growth factor, *TSP1* thrombospondin 1, *VEGF* vascular endothelial growth factor

To evaluate if MSC administration induce a provasculogenic microenvironment, the intraocular levels of pro-angiogenic and anti-angiogenic factors were measured. Four and 12 weeks after MSC administration, we did not observe differences in the mRNA levels of the pro-angiogenic factors VEGF-α, PDGF and ANG-1 in the eyes of DM + MSC mice compared to untreated DM mice (Fig. 9a). However, the mRNA level of TSP-1, a potent anti-angiogenic factor, was significantly increased in the eyes of DM + MSC mice compared to untreated DM mice and normal mice at both experimental times (Fig. 9a). The increase in TSP-1 level was also corroborated at protein level by ELISA (Fig. 9b). Additionally, retinal blood vessel density was evaluated in retinal flat-mounts after perfusion with high molecular weight FITC-dextran. At both experimental times, retinas of normal, DM and DM + MSC mice exhibited both superficial and deep vascular layers that extended from the optic nerve to the peripheral areas. The vessels formed a fine radial branching pattern in the superficial retinal layer and a polygonal reticular pattern in the deep retinal layer (Fig. 9c). There were no differences in the amount of blood vessels in the different experimental conditions (Fig. 9d).

An increase in vascular permeability is an early event in the onset of DR [44]. In our model, diabetes induced the appearance of areas of vascular leakage compared to normal mice. However, there was no difference in this parameter when comparing diabetic mice treated with vehicle or with MSCs, since in both cases we could detect the occasional presence of areas of vascular leakage (Additional file 4: Figure S3).

## Discussion

Diabetic retinopathy is a major cause of visual impairment and the leading cause of blindness in the Western world [2]. Stem cell-based therapy represents a newly emerging therapeutic approach to treat eye diseases. In this sense, due to the matching of the pathological events that occur at the initial stages of DR, and the cellular and molecular mechanisms associated with the reported MSC therapeutic effects, this approach represents a promising tool for the treatment of DR [44].

One of the first demonstrations that MSCs could play a therapeutic role in DR came from a study in which the intravenous administration of adipose-derived MSCs in an animal model of DM improved the integrity of the blood–retinal barrier [19]. However, in this study it was not clear whether the observed therapeutic effect was secondary to the reduced hyperglycemia, or due to a direct effect of MSCs in the damaged retina. Recently, more direct evidence of the role of MSCs in DR came from studies in which adipose-derived MSCs were intravitreally injected into the Akimba DR model or into diabetic rats treated with STZ [18, 26]. In these studies, the authors showed that MSC administration reduces blood–retinal barrier breakdown and MSCs differentiate into pericytes and integrate into the retinal vasculature [18, 26]. However, it has become more clear that DR affects not only retinal vasculature, but also retinal neuronal and glial cells, and that visual deficits in the early stage of DR are correlated with retinal neurosensory dysfunction that is mainly related to the early loss of RGCs [8, 45]. Thus, in this work we wanted to evaluate whether the intravitreal administration of MSCs was able to prevent the loss of RGCs. For this, we used an animal model of DM induced by the administration of a single high dose of STZ. This animal model is widely used to study the main complications associated with DM since animals develop sustained hyperglycemia and reduced insulinemia due to the massive destruction of pancreatic beta cells, allowing the appearance of the long-term complications associated with DM, including DR [28, 29].

In this animal model, as previously reported [10], diabetes induces a significant reduction in the number of RGCs. This 20 % reduction in the number of RGCs is in agreement with data reported for other animal models of DR [32, 46]. However, MSC administration completely prevents RGC loss, and this effect was maintained at least 12 weeks after the administration.

Both direct differentiation of MSCs into RGCs or indirect support to the neural retina by the secretion of neuroprotective factors or by the reduction of oxidative damage could be related to this therapeutic effect. Here we analyzed the migration of MSCs into the retina and the differentiation potential of these cells into RGCs, but also into astrocytes and pericytes since it has been reported the early damage to these cells could affect neuronal survival and the normal interactions with other retinal cells [47, 48]. We observed that most of the administered cells remained in the vitreous cavity, and they did not express neural or perivascular markers. We could detect only an extremely low number of MSCs that had adopted a putative phenotype of RGCs, astrocytes or pericytes, suggesting that the direct differentiation of MSCs into neural- or perivascular-like cells is not the main mechanism of the therapeutic effect. These results are in agreement with data of Johnson et al., who reported extremely poor retinal integration of MSCs after its intravitreal administration in an animal model of glaucoma [49]. In this sense, several authors have postulated that the pre-treatment of the retina with different drugs is needed to allow the effective incorporation of the transplanted cells [49, 50].

MSCs are known to produce and secrete, both in vitro and in vivo, a broad range of trophic factors, including some potent neurotrophic factors that could potentially prevent retinal neuronal cells from dying [51]. Here we saw that MSC administration increases the intraocular levels of NGF, GDNF and bFGF 4 and 12 weeks after injection. These neurotrophic factors have previously been involved in the inhibition of apoptosis of RGCs in different animal models [52, 53]. Therefore, MSCs remaining in the vitreous cavity could continuously deliver therapeutic molecules locally for a prolonged period of time. Alternatively, these neuroprotective factors could be secreted by the host retinal tissue in response to MSC injection.

Similar results were observed in an animal model of experimental glaucoma induced by the laser photocoagulation of the trabecular meshwork [21]. In this animal model, the intravitreal administration of MSCs provides trophic support to the damaged tissue, and increased RGC survival [21]. Additionally, Yu et al. reported that intravitreal MSC transplantation was neuroprotective after episcleral vein ligature, which can cause moderate ocular hypertension and RGC loss [54], and the same neuroprotective factors were also elevated in the vitreous cavity after the intravitreal administration of human placental stem cells in an animal model of DR [55].

In our model, MSC administration did not reduce the global apoptotic rate in the retina. However, we could not find RGCs undergoing apoptosis at any experimental time analyzed. This could be related to the slow onset and progression of DR, in which the number of cells dying at any given time is very small.

A widespread oxidative damage occurs in the retina of DR patients. Thus, in addition to providing neurotrophic support, MSCs may also give neuroprotection in the retina by modulating the levels of ROS. Previously we have demonstrated that MSCs have a high resistance to ROS due to the robust expression of SOD1, SOD2, CAT and GPX1 enzymes and high levels of glutathione [22]. Furthermore, MSCs constitutively express the enzymes required for the repair of oxidized structures [56]. Therefore, MSCs possess the main enzymatic machinery to detoxify ROS and to correct the oxidative damage. Here we showed that MSC administration induced a small reduction in the ROS level in the retina but a strong reduction in lipid peroxidation levels, one of the main markers of oxidative damage in the retina [57].

Consistent with these observations, the treatment of DM animals with antioxidants or with inhibitors of some of the metabolic pathways that generate ROS reduces oxidative damage of retinal structures and attenuates retinal cell loss [58–61]. Unfortunately, these drugs have low oral bioavailability or are not able to cross the blood–retinal barrier. Therefore large doses must be administrated to maintain therapeutic concentrations inside the retina, limiting their clinical use.

ERG is one of the most accepted techniques to evaluate retinal electrical response in diabetic individuals. In our animal model we observed a significant reduction in the amplitude of the scotopic a-wave and b-wave 3 months after diabetes induction that continued its altered state 4 and 6 months after STZ administration, but we could not detect changes in the amplitude of the OPs. MSC administration has a neutral effect on the electrical response of the retina. In our study we injected MSCs into the vitreous cavity, since it is the nearest location to RGCs, and in human patients intravitreal injection is technically feasible and safe [62]. However, it has been previously reported that cells injected into the vitreous cavity tend to cover the back of the lens and block the passage of light into the eye [21], altering the ERG analysis. Furthermore, we also observed that administered MSCs adopt this location, mainly between 7 and 30 days after its administration (data not shown). Therefore, another administration route, such as transplantation into the subretinal space, should be evaluated in order to avoid this effect.

Additionally, it has been previously shown that axon degeneration of the RGCs can occur with relative sparing of RGC bodies [63]; therefore, the soma protection achieved by the MSC administration in this context could not provide a functional benefit. Further investigation will be required to understand this effect.

One of the main concerns regarding MSC therapy for DR is the secretion of pro-angiogenic growth factors, mainly VEGF and PDGF, that might worsen the course of DR. VEGF is a potent growth factor that enhances vascular permeability, stimulates endothelial cell proliferation and migration, and promotes angiogenesis in the retina [61]. Similarly, PDGF is a pro-angiogenic growth factor that may also promote aberrant neovascularization in DR [64]. Furthermore, PDGF may stimulate the formation and traction of epiretinal membranes in patients with DR [65]. However, it has been reported that, depending on the microenvironment, MSCs could produce trophic factors that may modulate between a pro-angiogenic and anti-angiogenic environment. Here we showed that MSC administration did not increase the intraocular levels of VEGF and PDGF, and did not promote the massive formation of blood vessels or the increase in vascular permeability. Moreover, MSC administration increased the intraocular level of TSP-1, a potent anti-angiogenic factor. In accordance with our results, it has previously been shown that the local administration of MSCs in an animal model of corneal chemical injury, characterized by an accelerated neovascularization process, produced a rapid regression of the new blood vessels [66]. This anti-angiogenic effect was attributed to the secretion of TSP-1 by the administered MSCs, while the ocular levels of the pro-angiogenic factor VEGF were unchanged between the untreated and the MSC-treated group [66].

More studies are required in order to determine the best source and dose of MSCs and the optimal time for their administration. Nevertheless, the ability of MSCs to secrete neurotrophic factors and to reduce oxidative damage of the retina, preventing RGC loss, opens the possibility of using these cells to stop the progressive deterioration of the neural retina in diabetic individuals.

## Conclusion

Intravitreal administration of adipose-derived MSCs triggers an effective cytoprotective microenvironment in the retina of diabetic mice. Furthermore, MSC administration does not result in the pathological neovascularization of the retina. Thus, MSCs represent an interesting tool in order to prevent DR.

## Additional files

Additional file 1: Table S1. Primer and amplicon characteristics. (PNG 42 kb) Additional file 2: Figure S1. Donor MSCs differentiate into neural- or perivascular-like cells at very low frequency. One, 4, 8 and 12 weeks after MSC administration immunofluorescences were performed in eye sections to evaluate differentiation of MSCs into RGCs by colocalization of GFP with the ganglion cell marker β3-tubulin (A) or differentiation into astrocytes by colocalization of GFP with the astrocyte marker GFAP (B). To evaluate differentiation of MSCs into perivascular cells, immunofluorescences were performed to colocalize GFP with the pericyte marker NG2 in whole mount retinal preparations. Lectin was also added to detect blood vessels. Arrows indicate cells co-expressing both markers. Qualitative data are representative of six eyes per analyzed time. (PNG 2819 kb) Additional file 3: Figure S2. MSC administration does not modify the apoptotic rate in the retina of DM mice. Apoptosis in the retina was analyzed by the TUNEL technique in serial 4-μm eye sections 4 weeks after MSC administration (A) and 12 weeks after MSC administration (B). Nuclei were counterstained with DAPI. Arrows indicate TUNEL-positive cells. The number of apoptotic nuclei was quantified at both experimental times and expressed as fold-change versus normal mice (C and D). Qualitative data are representative of eight eyes per analyzed time. Quantitative data correspond to mean ± SEM of eight eyes per group and per analyzed time. (PNG 3862 kb) Additional file 4: Figure S3. MSC administration does not modify the presence of vascular leakage areas in the retina of DM mice. FITC-dextran was administered in the tail vein 4 and 12 weeks after the administration of MSCs or vehicle. Retinas were dissected, flat mounted and observed by confocal microscopy. Retinal vascular leakage was determined by the presence of areas of extravasated FITC-dextran marked by arrows. Qualitative data are representative of eight eyes per group and per analyzed time. (PNG 2029 kb)

## Abbreviations

α-MEM: α-Minimum essential medium
ANG-1: Angiopoietin 1
ANOVA: Analysis of variance
BDNF: Brain-derived neurotrophic factor
bFGF: Basic fibroblast growth factor
BHT: Butyl hydroxytoluene
DAPI: 4’-6’-Diamidino-2-phenylindole
DM: Diabetes mellitus
DR: Diabetic retinopathy
ELISA: Enzyme-linked immunosorbent assay
ERG: Electroretinography
FBS: Fetal bovine serum
GDNF: Glial cell line-derived neurotrophic factor
GFP: Green fluorescent protein
H_2_DCFDA: 2,7-Dichloro-dihydro-fluorescein diacetate
MDA: Malondialdehyde
MSC: Mesenchymal stromal cell
NGF: Nerve growth factor
OP: Oscillatory potential
PBS: Phosphate-buffered saline
PDGF: Platelet-derived growth factor
RGC: Retinal ganglion cell
ROS: Reactive oxygen species
RT-qPCR: Real-time quantitative polymerase chain reaction
SEM: Standard error of the mean
STZ: Streptozotocin
TSP-1: Thrombospondin-1
TUNEL: Terminal deoxynucleotidyl transferase (TdT)-mediated dUTP nick end labeling
VEGF: Vascular endothelial growth factor
