# Supplementary figures and images for: Intravitreal administration of multipotent mesenchymal stromal cells triggers a cytoprotective microenvironment in the retina of diabetic mice
Source: Stem Cell Res Ther. 2016 Mar 16;7:42. doi: 10.1186/s13287-016-0299-y (PMC4793534; doi:10.1186/s13287-016-0299-y)

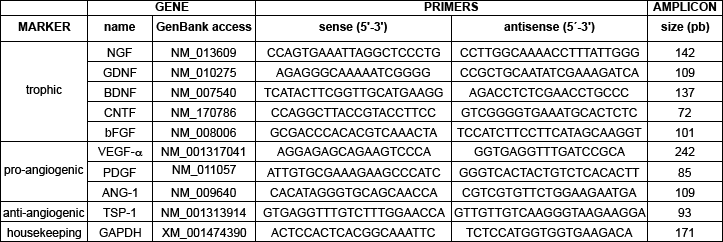

Supplement: Additional file 1: Table S1. — Primer and amplicon characteristics. (PNG 42 kb) [file 13287_2016_299_MOESM1_ESM.png]

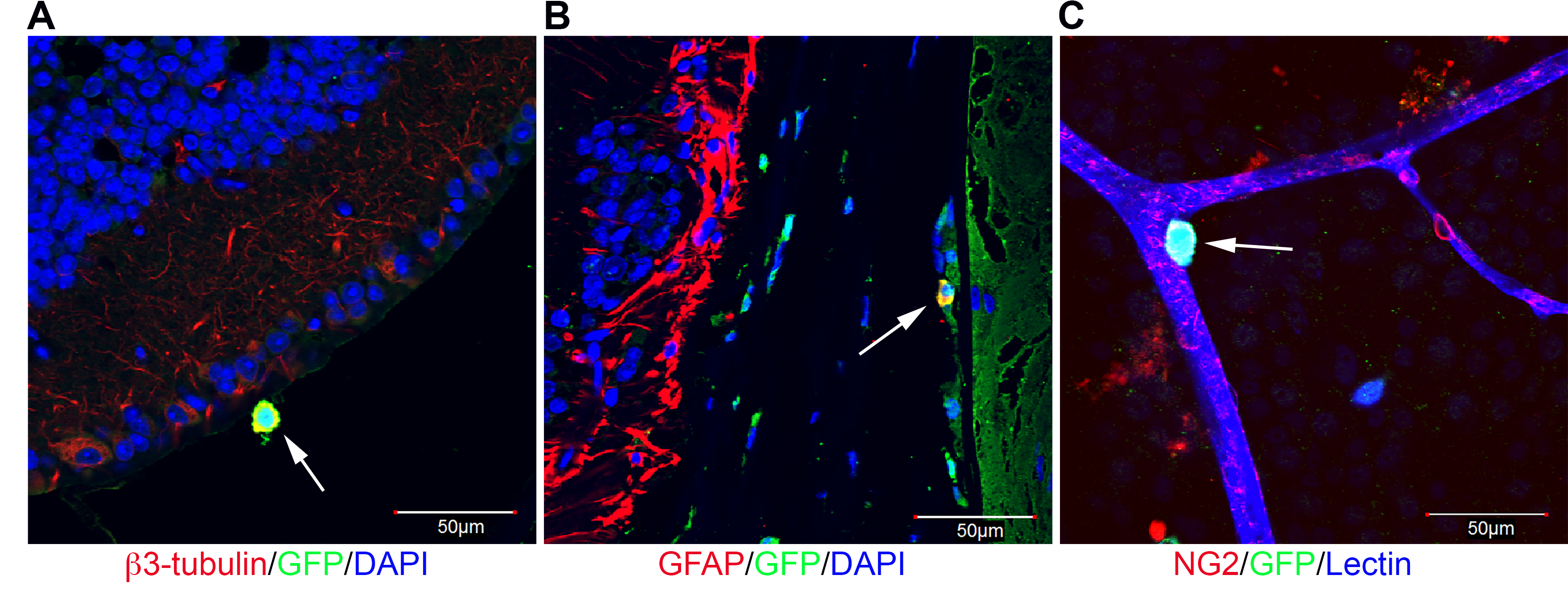

Supplement: Additional file 2: Figure S1. — Donor MSCs differentiate into neural- or perivascular-like cells at very low frequency. One, 4, 8 and 12 weeks after MSC administration immunofluorescences were performed in eye sections to evaluate differentiation of MSCs into RGCs by colocalization of GFP with the ganglion cell marker β3-tubulin (A) or differentiation into astrocytes by colocalization of GFP with the astrocyte marker GFAP (B). To evaluate differentiation of MSCs into perivascular cells, immunofluorescences were performed to colocalize GFP with the pericyte marker NG2 in whole mount retinal preparations. Lectin was also added to detect blood vessels. Arrows indicate cells co-expressing both markers. Qualitative data are representative of six eyes per analyzed time. (PNG 2819 kb) [file 13287_2016_299_MOESM2_ESM.png]

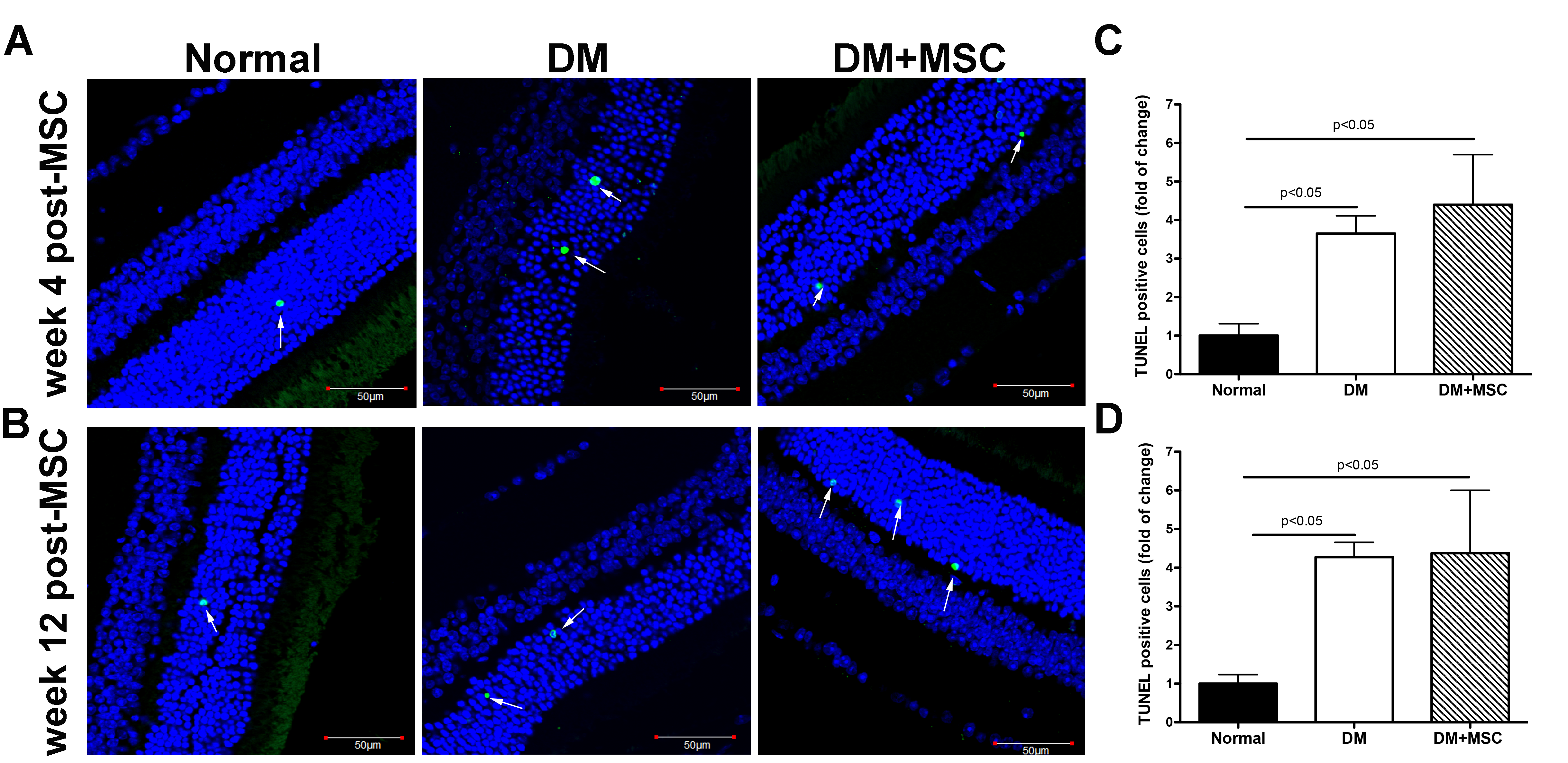

Supplement: Additional file 3: Figure S2. — MSC administration does not modify the apoptotic rate in the retina of DM mice. Apoptosis in the retina was analyzed by the TUNEL technique in serial 4-μm eye sections 4 weeks after MSC administration (A) and 12 weeks after MSC administration (B). Nuclei were counterstained with DAPI. Arrows indicate TUNEL-positive cells. The number of apoptotic nuclei was quantified at both experimental times and expressed as fold-change versus normal mice (C and D). Qualitative data are representative of eight eyes per analyzed time. Quantitative data correspond to mean ± SEM of eight eyes per group and per analyzed time. (PNG 3862 kb) [file 13287_2016_299_MOESM3_ESM.png]

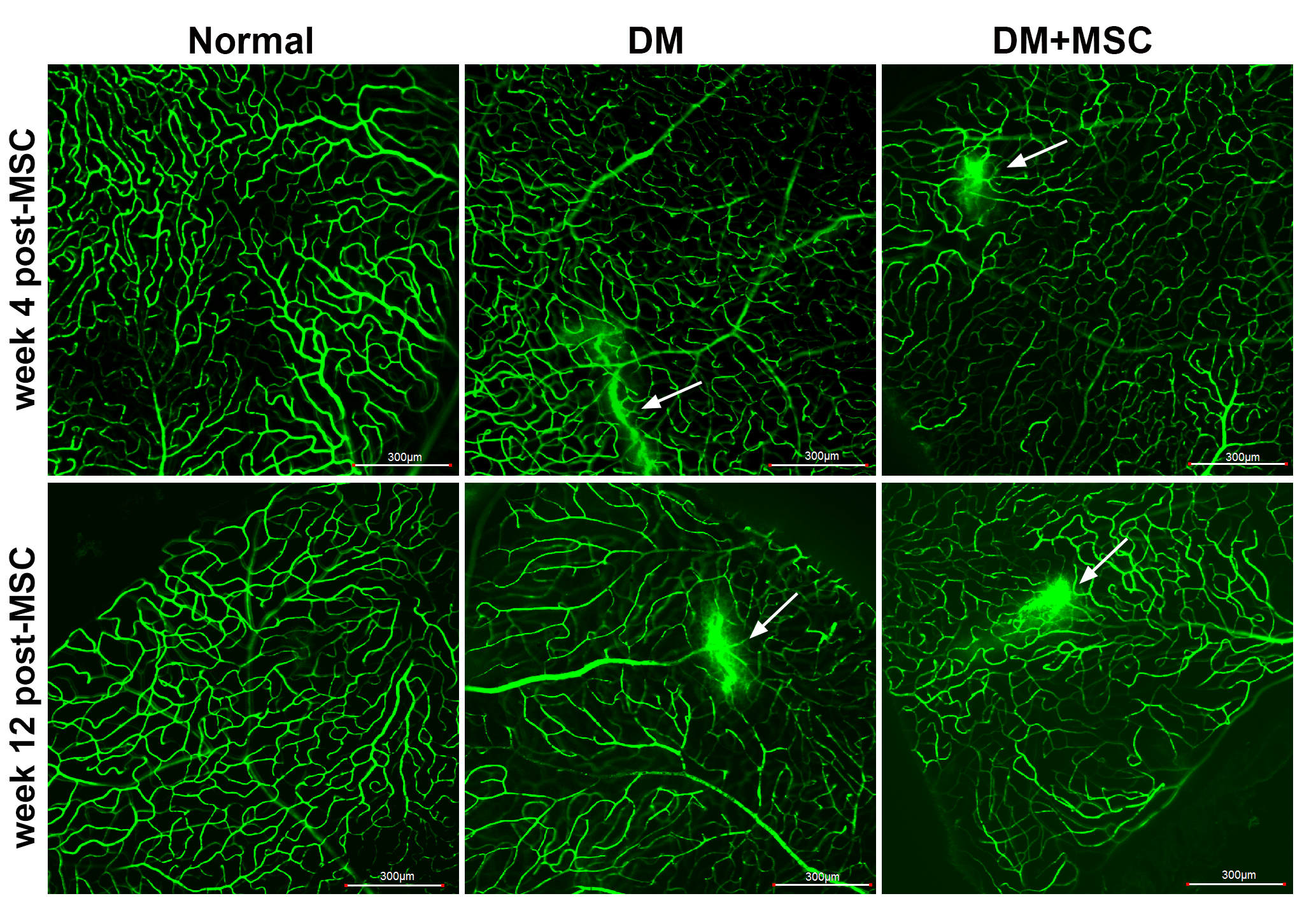

Supplement: Additional file 4: Figure S3. — MSC administration does not modify the presence of vascular leakage areas in the retina of DM mice. FITC-dextran was administered in the tail vein 4 and 12 weeks after the administration of MSCs or vehicle. Retinas were dissected, flat mounted and observed by confocal microscopy. Retinal vascular leakage was determined by the presence of areas of extravasated FITC-dextran marked by arrows. Qualitative data are representative of eight eyes per group and per analyzed time. (PNG 2029 kb) [file 13287_2016_299_MOESM4_ESM.png]
